# 4,4′,6,6′-Tetra­chloro-2,2′-[(1*E*,1′*E*)-propane-1,3-diylbis(nitrilo­methanylyl­idene)]diphenol

**DOI:** 10.1107/S1600536812029443

**Published:** 2012-07-04

**Authors:** Hadi Kargar, Reza Kia, Amir Adabi Ardakani, Muhammad Nawaz Tahir

**Affiliations:** a Department of Chemistry, Payame Noor University, PO BOX 19395-3697 Tehran, I. R. of IRAN; b Department of Chemistry, Science and Research Branch, Islamic Azad University, Tehran, Iran; c Department of Physics, University of Sargodha, Punjab, Pakistan

## Abstract

The title compound, C_17_H_14_Cl_4_N_2_O_2_, is generated by crystallographic twofold symmetry. The two benzene rings are inclined to one another by 80.17 (10)°. There are two intra­molecular O—H⋯N hydrogen bonds, which make *S*(6) ring motifs. In the crystal, mol­ecules are linked by C—H⋯O and weak C—H⋯Cl inter­actions, forming a three-dimensional network.

## Related literature

For standard bond lengths, see: Allen *et al.*, (1987 ▶). For hydrogen-bond motifs, see: Bernstein *et al.* (1995 ▶). For related Schiff base ligands, see: Kargar *et al.* (2011 ▶); Kia *et al.* (2010 ▶).
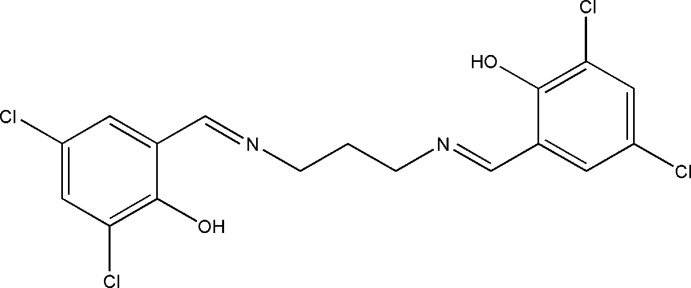


## Experimental

### 

#### Crystal data

C_17_H_14_Cl_4_N_2_O_2_
*M* *_r_* = 420.10
Orthorhombic, 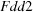

*a* = 24.9797 (14) Å
*b* = 31.666 (3) Å
*c* = 4.4495 (2) Å
*V* = 3519.6 (4) Å^3^
*Z* = 8
Mo *K*α radiation
μ = 0.69 mm^−1^
*T* = 291 K
0.26 × 0.23 × 0.18 mm

#### Data collection

Bruker SMART APEXII CCD area-detector diffractometer
Absorption correction: multi-scan (*SADABS*; Bruker, 2005 ▶) *T*_min_ = 0.842, *T*_max_ = 0.886
7926 measured reflections
1960 independent reflections
1634 reflections with *I* > 2σ(*I*)
*R*_int_ = 0.028

#### Refinement

*R*[*F* ^2^ > 2σ(*F* ^2^)] = 0.027
*wR*(*F* ^2^) = 0.068
*S* = 1.04
1960 reflections
115 parameters
1 restraint
H-atom parameters constrained
Δρ_max_ = 0.14 e Å^−3^
Δρ_min_ = −0.16 e Å^−3^
Absolute structure: Flack (1983 ▶), 842 Friedel pairs
Flack parameter: 0.08 (7)

### 

Data collection: *APEX2* (Bruker, 2005 ▶); cell refinement: *SAINT* (Bruker, 2005 ▶); data reduction: *SAINT*; program(s) used to solve structure: *SHELXS97* (Sheldrick, 2008 ▶); program(s) used to refine structure: *SHELXL97* (Sheldrick, 2008 ▶); molecular graphics: *SHELXTL* (Sheldrick, 2008 ▶); software used to prepare material for publication: *SHELXTL* and *PLATON* (Spek, 2009 ▶).

## Supplementary Material

Crystal structure: contains datablock(s) global, I. DOI: 10.1107/S1600536812029443/su2464sup1.cif

Structure factors: contains datablock(s) I. DOI: 10.1107/S1600536812029443/su2464Isup2.hkl

Additional supplementary materials:  crystallographic information; 3D view; checkCIF report

## Figures and Tables

**Table 1:** Hydrogen-bond geometry (Å, °)

*D*—H⋯*A*	*D*—H	H⋯*A*	*D*⋯*A*	*D*—H⋯*A*
O1—H1⋯N1	0.82	1.84	2.574 (2)	147
C5—H2⋯O1^i^	0.93	2.43	3.336 (2)	166
C8—H5*B*⋯Cl1^ii^	0.97	2.89	3.851 (2)	169

Symmetry codes: (i) 
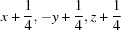
; (ii) 
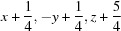
.

**Fig. 1.**
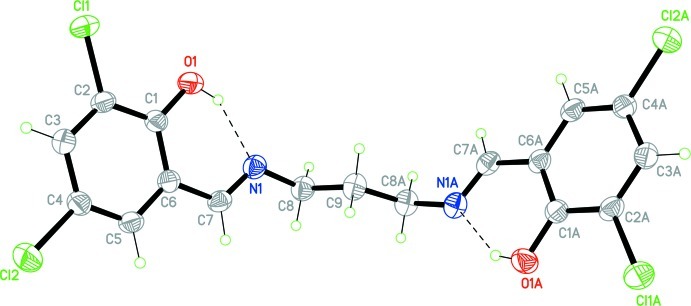
The molecular structure of the title compound, showing 40% probability displacement ellipsoids and the atomic numbering [symmetry code for suffix A = -x, -y, z].

**Fig. 2.**
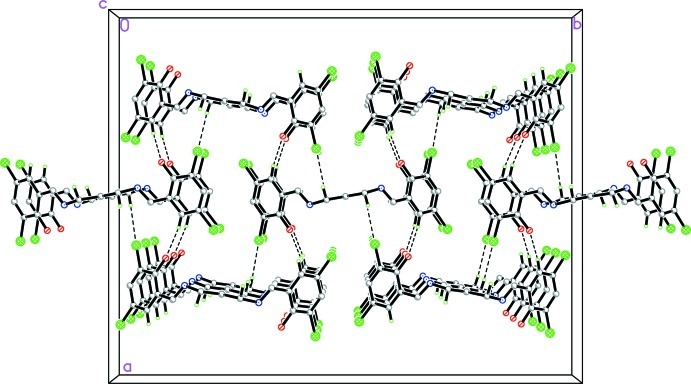
The crystal packing diagram of the title compound viewed down the c-axis, showing linking of molecules through C—H···O and weak C—H···Cl interactions (dashed lines).

## supplementary crystallographic information

### Comment

In continuation of our work on the crystal structure analyses of Schiff base ligands (Kargar *et al.*, (2011); Kia *et al.*, (2010), we synthesized the title compound and report herein on its crystal structure.

The title compound, Fig. 1, a potential tetradentate Schiff base ligand, possesses two-fold rotation symmetry, atom C9 is located on the 2-fold axis. The bond lengths (Allen *et al.*, 1987) and angles are within the normal ranges. The two symmetry related benzene rings are inclined to one another by 80.17 (10) °. There are two intramolecular O—H···N hydrogen bonds which make *S(6)* ring motifs (Table 1; Bernstein *et al.*, 1995).

In the crystal, molecules are linked by C—H···O and weak C—H···Cl interactions to form a three-dimensional network (Table 1 and Fig. 2).

### Experimental

The title compound was synthesized by adding 3,5-dichlorosalicylaldehyde (2 mmol) to a solution of propylenediamine (1 mmol) in ethanol (30 ml). The mixture was refluxed with stirring for 30 min. The resultant solution was filtered. Light-yellow prismatic single crystals of the title compound, suitable for *X*-ray structure determination, were recrystallized from ethanol by slow evaporation of the solvents at room temperature over several days.

### Refinement

The OH and C-bound H atoms were included in calculated positions and treated as riding atoms: O-H = 0.82 Å, C-H = 0.93 and 0.96 Å, with U_iso_(H) = k × U_eq_(O,C) where k = 1.5 for OH and CH_3_ H atoms and = 1.2 for other H atoms.

### Crystal data

**Table:**

C_17_H_14_Cl_4_N_2_O_2_	*F*(000) = 1712
*M**_r_* = 420.10	*D*_x_ = 1.586 Mg m^−^^3^
Orthorhombic, *F**d**d*2	Mo *K*α radiation, λ = 0.71073 Å
Hall symbol: F 2 -2d	Cell parameters from 1234 reflections
*a* = 24.9797 (14) Å	θ = 2.5–27.5°
*b* = 31.666 (3) Å	µ = 0.69 mm^−^^1^
*c* = 4.4495 (2) Å	*T* = 291 K
*V* = 3519.6 (4) Å^3^	Prism, light-yellow
*Z* = 8	0.26 × 0.23 × 0.18 mm

### Data collection

**Table:**

Bruker SMART APEXII CCD area-detector diffractometer	1960 independent reflections
Radiation source: fine-focus sealed tube	1634 reflections with *I* > 2σ(*I*)
Graphite monochromator	*R*_int_ = 0.028
φ and ω scans	θ_max_ = 27.3°, θ_min_ = 2.1°
Absorption correction: multi-scan (*SADABS*; Bruker, 2005)	*h* = −32→32
*T*_min_ = 0.842, *T*_max_ = 0.886	*k* = −40→40
7926 measured reflections	*l* = −5→5

### Refinement

**Table:**

Refinement on *F*^2^	Secondary atom site location: difference Fourier map
Least-squares matrix: full	Hydrogen site location: inferred from neighbouring sites
*R*[*F*^2^ > 2σ(*F*^2^)] = 0.027	H-atom parameters constrained
*wR*(*F*^2^) = 0.068	*w* = 1/[σ^2^(*F*_o_^2^) + (0.0306*P*)^2^ + 1.6825*P*] where *P* = (*F*_o_^2^ + 2*F*_c_^2^)/3
*S* = 1.04	(Δ/σ)_max_ < 0.001
1960 reflections	Δρ_max_ = 0.14 e Å^−^^3^
115 parameters	Δρ_min_ = −0.16 e Å^−^^3^
1 restraint	Absolute structure: Flack (1983), 842 Friedel pairs
Primary atom site location: structure-invariant direct methods	Flack parameter: 0.08 (7)

### Special details

**Table:**

Geometry. All esds (except the esd in the dihedral angle between two l.s. planes) are estimated using the full covariance matrix. The cell esds are taken into account individually in the estimation of esds in distances, angles and torsion angles; correlations between esds in cell parameters are only used when they are defined by crystal symmetry. An approximate (isotropic) treatment of cell esds is used for estimating esds involving l.s. planes.
Refinement. Refinement of F^2^ against ALL reflections. The weighted R-factor wR and goodness of fit S are based on F^2^, conventional R-factors R are based on F, with F set to zero for negative F^2^. The threshold expression of F^2^ > 2sigma(F^2^) is used only for calculating R-factors(gt) etc. and is not relevant to the choice of reflections for refinement. R-factors based on F^2^ are statistically about twice as large as those based on F, and R- factors based on ALL data will be even larger.

### Fractional atomic coordinates and isotropic or equivalent isotropic displacement parameters (Å^2^)

**Table:**

	*x*	*y*	*z*	*U*_iso_*/*U*_eq_	Occ. (<1)
C1	−0.04810 (7)	0.14212 (6)	0.7828 (4)	0.0378 (4)	
C2	−0.05597 (7)	0.17577 (6)	0.5854 (5)	0.0405 (5)	
C3	−0.01420 (7)	0.19985 (6)	0.4810 (5)	0.0429 (5)	
H9	−0.0203	0.2218	0.3466	0.052*	
C4	0.03720 (8)	0.19078 (6)	0.5799 (5)	0.0419 (5)	
C5	0.04673 (7)	0.15902 (6)	0.7793 (5)	0.0409 (5)	
H2	0.0814	0.1538	0.8453	0.049*	
C6	0.00437 (7)	0.13440 (6)	0.8845 (4)	0.0374 (4)	
C7	0.01466 (8)	0.10036 (6)	1.0958 (4)	0.0401 (5)	
H4	0.0489	0.0972	1.1753	0.048*	
C8	−0.00878 (9)	0.03959 (6)	1.3730 (4)	0.0458 (5)	
H5A	−0.0378	0.0351	1.5143	0.055*	
H5B	0.0234	0.0461	1.4863	0.055*	
C9	0.0000	0.0000	1.1876 (7)	0.0465 (7)	
H6A	−0.0309	−0.0043	1.0589	0.056*	0.50
H6B	0.0309	0.0043	1.0589	0.056*	0.50
Cl1	−0.12035 (2)	0.187207 (19)	0.46609 (15)	0.05937 (17)	
Cl2	0.09006 (2)	0.220654 (18)	0.43888 (16)	0.06188 (18)	
N1	−0.02186 (7)	0.07479 (5)	1.1743 (4)	0.0424 (4)	
O1	−0.08951 (5)	0.11861 (4)	0.8692 (4)	0.0503 (4)	
H1	−0.0790	0.0999	0.9826	0.075*	

### Atomic displacement parameters (Å^2^)

**Table:**

	*U*^11^	*U*^22^	*U*^33^	*U*^12^	*U*^13^	*U*^23^
C1	0.0349 (9)	0.0355 (9)	0.0429 (12)	−0.0002 (8)	−0.0034 (8)	−0.0052 (9)
C2	0.0322 (9)	0.0401 (10)	0.0490 (12)	0.0056 (8)	−0.0083 (8)	−0.0044 (9)
C3	0.0415 (10)	0.0356 (9)	0.0518 (13)	0.0015 (8)	−0.0029 (10)	0.0001 (10)
C4	0.0349 (10)	0.0359 (10)	0.0550 (13)	−0.0016 (8)	0.0015 (9)	−0.0067 (9)
C5	0.0314 (9)	0.0402 (10)	0.0512 (13)	0.0052 (8)	−0.0060 (8)	−0.0087 (9)
C6	0.0368 (9)	0.0344 (9)	0.0409 (11)	0.0028 (7)	−0.0035 (8)	−0.0085 (9)
C7	0.0374 (10)	0.0420 (11)	0.0410 (11)	0.0067 (8)	−0.0083 (8)	−0.0084 (9)
C8	0.0521 (12)	0.0446 (11)	0.0407 (12)	0.0032 (9)	−0.0053 (9)	−0.0001 (10)
C9	0.0567 (17)	0.0430 (15)	0.0399 (15)	0.0048 (13)	0.000	0.000
Cl1	0.0371 (3)	0.0610 (3)	0.0800 (4)	0.0024 (2)	−0.0162 (3)	0.0145 (3)
Cl2	0.0434 (3)	0.0532 (3)	0.0891 (5)	−0.0076 (2)	0.0080 (3)	0.0055 (3)
N1	0.0464 (9)	0.0411 (9)	0.0397 (9)	0.0039 (7)	−0.0062 (8)	−0.0015 (8)
O1	0.0366 (7)	0.0503 (9)	0.0639 (10)	−0.0058 (6)	−0.0093 (6)	0.0124 (7)

### Geometric parameters (Å, º)

**Table:**

C1—O1	1.331 (2)	C6—C7	1.453 (3)
C1—C2	1.395 (3)	C7—N1	1.269 (2)
C1—C6	1.408 (2)	C7—H4	0.9300
C2—C3	1.373 (3)	C8—N1	1.460 (3)
C2—Cl1	1.7318 (19)	C8—C9	1.517 (3)
C3—C4	1.387 (3)	C8—H5A	0.9700
C3—H9	0.9300	C8—H5B	0.9700
C4—C5	1.362 (3)	C9—C8^i^	1.517 (3)
C4—Cl2	1.741 (2)	C9—H6A	0.9700
C5—C6	1.395 (3)	C9—H6B	0.9700
C5—H2	0.9300	O1—H1	0.8200
			
O1—C1—C2	119.98 (16)	N1—C7—C6	121.61 (17)
O1—C1—C6	122.23 (17)	N1—C7—H4	119.2
C2—C1—C6	117.79 (17)	C6—C7—H4	119.2
C3—C2—C1	122.02 (17)	N1—C8—C9	109.52 (18)
C3—C2—Cl1	119.06 (15)	N1—C8—H5A	109.8
C1—C2—Cl1	118.92 (15)	C9—C8—H5A	109.8
C2—C3—C4	118.75 (19)	N1—C8—H5B	109.8
C2—C3—H9	120.6	C9—C8—H5B	109.8
C4—C3—H9	120.6	H5A—C8—H5B	108.2
C5—C4—C3	121.42 (18)	C8^i^—C9—C8	114.1 (3)
C5—C4—Cl2	120.22 (15)	C8^i^—C9—H6A	108.7
C3—C4—Cl2	118.35 (16)	C8—C9—H6A	108.7
C4—C5—C6	119.90 (17)	C8^i^—C9—H6B	108.7
C4—C5—H2	120.1	C8—C9—H6B	108.7
C6—C5—H2	120.1	H6A—C9—H6B	107.6
C5—C6—C1	120.07 (18)	C7—N1—C8	119.53 (17)
C5—C6—C7	119.82 (17)	C1—O1—H1	109.5
C1—C6—C7	120.10 (18)		
			
O1—C1—C2—C3	−177.41 (19)	C4—C5—C6—C7	179.60 (17)
C6—C1—C2—C3	2.7 (3)	O1—C1—C6—C5	177.77 (18)
O1—C1—C2—Cl1	2.0 (2)	C2—C1—C6—C5	−2.4 (3)
C6—C1—C2—Cl1	−177.84 (15)	O1—C1—C6—C7	−1.3 (3)
C1—C2—C3—C4	−1.2 (3)	C2—C1—C6—C7	178.59 (16)
Cl1—C2—C3—C4	179.35 (16)	C5—C6—C7—N1	−173.18 (19)
C2—C3—C4—C5	−0.7 (3)	C1—C6—C7—N1	5.9 (3)
C2—C3—C4—Cl2	178.41 (15)	N1—C8—C9—C8^i^	−174.57 (19)
C3—C4—C5—C6	1.0 (3)	C6—C7—N1—C8	176.32 (17)
Cl2—C4—C5—C6	−178.09 (15)	C9—C8—N1—C7	−97.64 (19)
C4—C5—C6—C1	0.5 (3)		

Symmetry code: (i) −*x*, −*y*, *z*.

### Hydrogen-bond geometry (Å, º)

**Table:**

*D*—H···*A*	*D*—H	H···*A*	*D*···*A*	*D*—H···*A*
O1—H1···N1	0.82	1.84	2.574 (2)	147
C5—H2···O1^ii^	0.93	2.43	3.336 (2)	166
C8—H5*B*···Cl1^iii^	0.97	2.89	3.851 (2)	169

Symmetry codes: (ii) *x*+1/4, −*y*+1/4, *z*+1/4; (iii) *x*+1/4, −*y*+1/4, *z*+5/4.
